# Mortality limits used in wind energy impact assessment underestimate impacts of wind farms on bird populations

**DOI:** 10.1002/ece3.6360

**Published:** 2020-06-04

**Authors:** Peter Schippers, Ralph Buij, Alex Schotman, Jana Verboom, Henk van der Jeugd, Eelke Jongejans

**Affiliations:** ^1^ Wageningen Environmental Research Wageningen University & Research Wageningen The Netherlands; ^2^ Environmental Systems Analysis Wageningen University Wageningen The Netherlands; ^3^ Vogeltrekstation – Dutch Centre for Avian Migration and Demography (NIOO‐KNAW) Wageningen The Netherlands; ^4^ Animal Ecology and Physiology Radboud University Nijmegen The Netherlands

**Keywords:** bird mortality, collisions, Ornis 1% mortality criterion, population viability, potential biological removal, threshold assessment methods, wind farm

## Abstract

1. The consequences of bird mortality caused by collisions with wind turbines are increasingly receiving attention. So‐called acceptable mortality limits of populations, that is, those that assume that 1%–5% of additional mortality and the potential biological removal (PBR), provide seemingly clear‐cut methods for establishing the reduction in population viability.
2. We examine how the application of these commonly used mortality limits could affect populations of the Common Starling, Black‐tailed Godwit**,** Marsh Harrier, Eurasian Spoonbill, White Stork, Common Tern, and White‐tailed Eagle using stochastic density‐independent and density‐dependent Leslie matrix models.
3. Results show that population viability can be very sensitive to proportionally small increases in mortality. Rather than having a negligible effect, we found that a 1% additional mortality in postfledging cohorts of our studied populations resulted in a 2%–24% decrease in the population level after 10 years. Allowing a 5% mortality increase to existing mortality resulted in a 9%–77% reduction in the populations after 10 years.
4. When the PBR method is used in the density‐dependent simulations, the proportional change in the resulting growth rate and carrying capacity was species‐independent and largely determined by the recovery factor (*F*_r_). When *F*_r_ = 1, a value typically used for robust populations, additional mortality resulted in a 50%–55% reduction in the equilibrium density and the resulting growth rate. When *F*_r_ = 0.1, used for threatened populations, the reduction in the equilibrium density and growth rate was about 5%.
5. *Synthesis and applications*. Our results show that by allowing a mortality increase from wind farm collisions according to both criteria, the population impacts of these collisions can still be severe. We propose a simple new method as an alternative that was able to estimate mortality impacts of age‐structured stochastic density‐dependent matrix models.

## Graphical abstract

In this paper, we evaluate two commonly used acceptable mortality limits that should safeguard bird population from wind turbine collision. We use age‐structured matrix models of birds (11 cases) with contrasting age of first reproduction to evaluate these mortality limits. Results show that populations can still suffer severe losses by using these approaches. Alternatively, we developed a robust and a simple model that summarizes the expected population reduction in the matrix models.

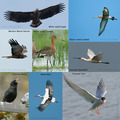

## 1 INTRODUCTION

The consequences of additional mortality of birds from collisions with a rapidly increasing number of wind turbines are receiving attention worldwide (Marques et al., 2014; Schuster, Bulling, & Koppel, 2015). Wind turbine collisions have proven to affect bird populations, with potentially important negative, cumulative effects from additional mortality caused by multiple wind farms (Bellebaum, Korner‐Nievergelt, Durr, & Mammen, 2013; Drewitt & Langston, 2006, 2008). Given these impacts of wind turbines on bird populations, the European Union (EU) introduced procedures to ensure that wind development projects comply with the protection measures and the precautionary principle enshrined in the EU Birds and Habitats Directives (Directive 2009/147/EC and Council Directive 92/43/EEC). Together, these dictate that populations of naturally occurring wild bird species present in the EU are maintained or restored at a level that will ensure their long‐term survival and so‐called “favorable conservation status.” Planned wind farms possibly harming these goals therefore have to undergo a step‐by‐step impact assessment procedure and, where necessary, apply the relevant safeguards for the species and habitat types of EU interest.

The general system of protection in the EU prohibits deliberate killing, capture, or disturbance, which, according to jurisprudence, is the case if birds are killed as a result of collision with wind turbines (De Sateleer, 2013). However, member states may derogate from the provisions on species “to permit, under strictly supervised conditions and on a selective basis, the capture, keeping or other judicious use of certain birds in small numbers” (art. 9(1)(c) of the Council Directive 79/409/EEC of 2 April 1979 on the conservation of wild birds). The threshold below which the derogation is automatically considered as meeting the requirements of the notion of “small numbers” is currently set at 1% of the overall annual natural mortality in the relevant biogeographical population. This derogation is the origin of the so‐called “1% mortality criterion” as developed by the ORNIS committee (European‐Commission, 1993). This criterion is said to meet the condition of a negligible effect on the population dynamics of the species concerned, considering the fact that the mortality parameters are often not known with an accuracy of 1%. In the Netherlands and Belgium, the ORNIS criterion is often used to determine whether any additional mortality might have a significant impact on a population of a particular species affected by wind turbine collisions (Backes & Akerboom, 2018). For abundant species with a favorable conservation status, the threshold is set higher at a maximum of 5% additional mortality in Belgium, while the ORNIS criterion is often applied irrespective of the species status and its population size in the Netherlands. Elsewhere, other threshold measures are used, such as the so‐called “Mortalitäts‐Gefährdungs‐Index” in Germany which uses a “significance” threshold in the range of 0.5%–5% additional mortality (Dierschke & Bernotat, 2018).

In addition to additional mortality thresholds, the potential biological removal (PBR) method is widely used to define a level of acceptable extra mortality, or “harvest,” that a population can tolerate (Wade, 1998). When used in conjunction with the ORNIS criterion, the latter often functions as a first, rough estimate after which the PBR method is applied in case the 1% additional mortality threshold is exceeded. The PBR method identifies a threshold of additional mortality below which a decline of the affected population to eventual extinction would be unlikely (Niel & Lebreton, 2005). The PBR provides thresholds of additional mortality that account for the growth rate at low densities and assumes density‐dependent effects on the population. The PBR threshold for any population includes a so‐called “recovery factor” *F*_r_ (0.1–1), which provides a safety margin for species vulnerability (Dillingham & Fletcher, 2008).

In addition to collision rates, population size and the timing of collisions are key to determining the species‐specific vulnerability to wind farm collisions (Drewitt & Langston, 2006). In general, the consequences of wind turbine mortality for any bird population depend on that population's ability to compensate for increases in mortality rates through density‐dependent processes (Liley & Sutherland, 2007; Newton, 1998). In populations that are strongly limited by density dependence, the loss of any individual might be compensated by the increased fitness of the remainder of the individuals in the population, for example, through an increase in the average territory quality (Matthysen, 1990) or per capita food availability (Martin, 1987). Long‐lived species with low reproductive rates are likely to be more sensitive to an increase in adult mortality and less able to compensate by increasing reproduction (Saether & Bakke, 2000), which explains why even low collision rates may significantly contribute to population declines or demographic changes in various long‐lived species (Schaub, 2012).

The mortality thresholds commonly used to evaluate the expected effect of wind turbines on local bird populations are convenient to decision makers because they offer an apparently science‐based and clear‐cut method to establish whether damage to the integrity of a population will or will not occur. However, questions have recently been raised as to the validity of their use (Green, Langston, McCluskie, Sutherland, & Wilson, 2016; Horswill, O'Brien, & Robinson, 2017). Such questions refer to the general applicability of the threshold assessments, notably their uncertainty with regard to predicting impacts of additional mortality on species with different life‐history strategies, as well as the cumulative impacts of wind turbine collision on population developments. A key criticism made by Green et al. (2016), and supported by the conclusions of Horswill et al. (2017), was that the assumed density‐dependent processes operating on bird populations may be highly uncertain, generating misleading conclusions regarding the impact of additional mortality when such assumptions are not met (O'Brien, Cook, & Robinson, 2017).

**FIGURE 1:**
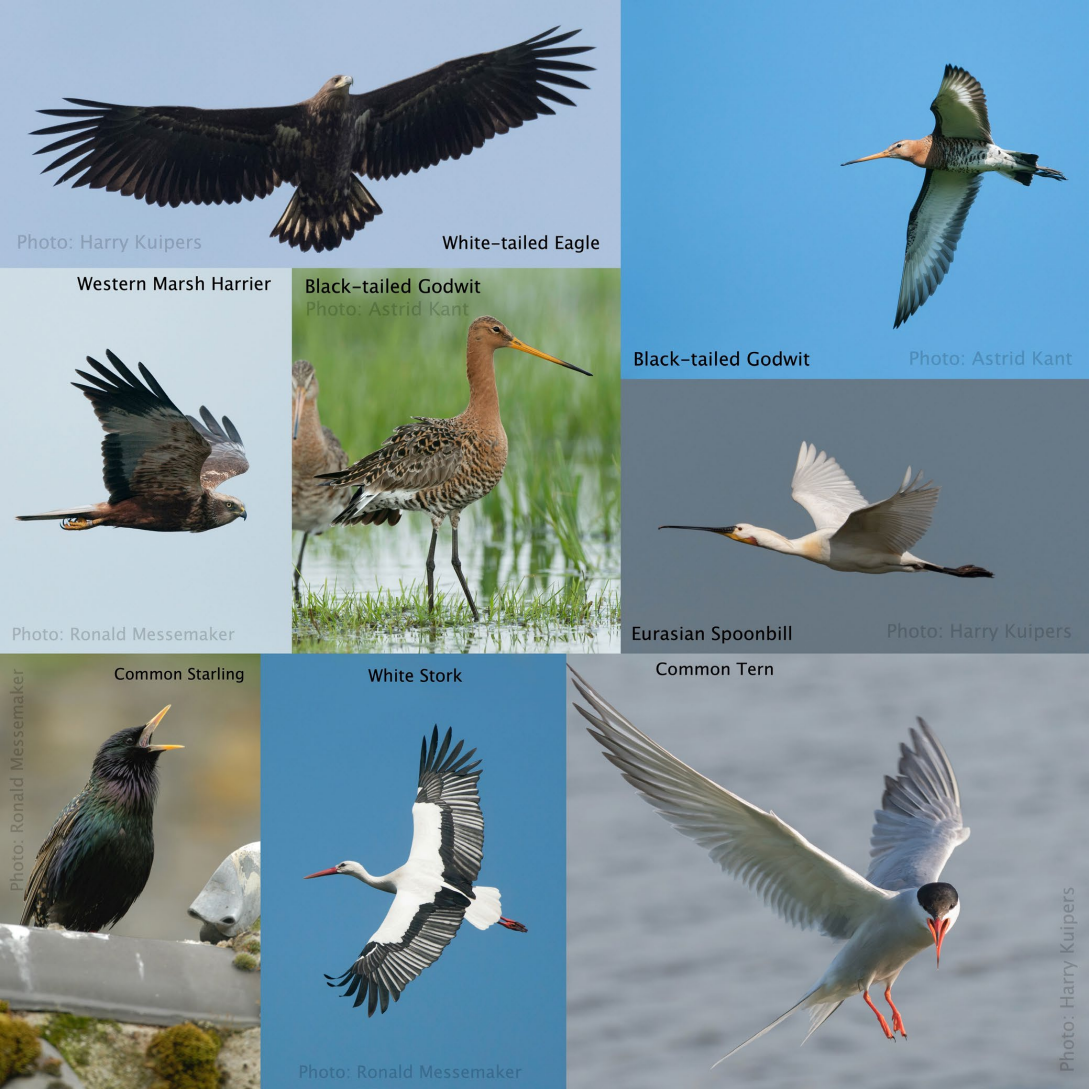
Bird species studied in this paper

Given these considerations, we investigate how populations of bird species with different life histories that are also prone to wind turbine collision are affected by mortality thresholds used in wind energy impact assessment. We consider populations of the following species: Common Starling (*Sturnus vulgaris*), Black‐tailed Godwit (*Limosa limosa*), Eurasian Spoonbill (*Pilatalea leucorodia*), Western Marsh Harrier (*Circus aeruginosus*), White Stork (*Ciconia ciconia*), Common Tern (*Sterna hirundo*), and White‐tailed Eagle (*Haliaeetus albicilla*; Figure 1). Because it is uncertain whether the dynamics studied populations are strongly regulated by density‐dependent processes, we use both density‐dependent and density‐independent population models. We address the following three questions.

First, what is the impact of a small mortality increase (e.g., 1%–5% of natural mortality) on the population viability of density‐independent populations? Because these mortality thresholds are destined to safeguard populations (Backes & Akerboom, 2018; Dierschke & Bernotat, 2018), we hypothesize that small mortality increases only have a limited impact on the viability of populations.

Second, what is the impact of a small mortality increase and harvesting based on potential biological removal (PBR) on the dynamics of density‐dependent populations? We expect density dependence to compensate for the loss caused by wind farms (Liley & Sutherland, 2007; Newton, 1998). Therefore, we hypothesized that a small increase in mortality, defined as % additional mortality and PBR, has a negligible effect on the viability of density‐dependent populations. However, when populations suffer accumulative additional mortality from multiple wind farms to higher mortality rates (e.g., 10% additional mortality), we expect a more significant impact on population viability.

Third, are populations of long‐lived species (with high age of first reproduction) more vulnerable to extra mortality than short‐lived species? We hypothesized that populations of long‐lived species are more sensitive to wind turbine collision because they are less able to compensate by increasing reproduction (Saether & Bakke, 2000).

## 2 MATERIALS AND METHODS

### 2.1 Parameterization of bird population models

In order to assess the effects of extra mortality upon bird populations, we used Leslie matrix population models with a one‐year time step. These age‐structured matrix models were parameterized with species‐specific survival and reproduction rates. We selected populations of seven species based on the availability of data, considerable likelihood to collide with wind turbines, and contrasting ages of first reproduction. For species for which long time series of demographic data were available with population trends clearly changing over time, we separately assessed periods with contrasting population trends, as detailed in the species descriptions below. Mean survival and reproduction rates, standard deviations, and additional information like the age of first reproduction can be found in Table 1. R code detailing how these estimates are used to construct age‐structured population matrix models is deposited in the data repository (see Data Availability Statement). In these so‐called postbreeding census models, the birds in the first class were 0 years old (Caswell, 2001).

**TABLE 1:** Vital rates, growth rate and elasticity of seven bird species at various locations and periods ± standard deviation

Vital rates	Common Starling			Black‐tailed Godwit		Marsh Harrier	Spoonbill	White Stork	Common Tern	White‐tailed Eagle	
Period (year)	1960–1978 (19)	1978–1990 (12)	1990–2012 (21)	2011–2016 (5)	2012–2016 (4)	1997–2015 (19)	1994–2008 (15)	1977–2000 (23)	1994–2008 (10)	1947–1974 (26)	1975–2008 (34)
Country	The Netherlands	The Netherlands	The Netherlands	The Netherlands	The Netherlands	The Netherlands	The Netherlands	Switzerland	The Netherlands	Germany	Germany
Region	The Netherlands	The Netherlands	The Netherlands	Kuststrook	Skriezekrite	The Netherlands	The Netherlands	Switzerland	IJsselmeer	Schlesw.‐Holst.	Schlesw.‐Holst.
Age of first reproduction	1	1	1	2	2	3	3	3	4	5	5
# Fledglings per breeding pair	2.56 ± 0	4.17 ± 0.238	3.73 ± 0.546	1.800 ± 0.618	2.461 ± 0.787	2.124 ± 0.409	1.859 ± 0.696	1.560 ± 0.463	1.294 ± 1.024	0.454 ± 0.278	1.573 ± 0.387
First‐year survival	0.331 ± 0.035	0.181 ± 0.051	0.102 ± 0.034	0.169 ± 0.074	0.169 ± 0.113	0.641 ± 0.093	0.607 ± 0.126	0.390 ± 0.096	0.555 ± 0.186	0.720	0.741
Second‐year survival	0.677 ± 0.049	0.615 ± 0.039	0.607 ± 0.151	0.858 ± 0.007	0.859 ± 0.007	0.804 ± 0.063	0.893 ± 0.026	0.861 ± 0.047	0.588 ± 0.208	0.889	0.800
Annual survival older birds	0.677 ± 0.049	0.615 ± 0.039	0.607 ± 0.151	0.858 ± 0.007	0.859 ± 0.007	0.804 ± 0.063	0.877 ± 0.010	0.861 ± 0.047	0.724 ± 0.131	0.816^a^	0.813^a^
Probability of adults breeding	1.0	1.0	1.0	1.0	1.0	0.5	0.63–0.95	0.475–1.0	1.0	0.954	0.954
Growth rate matrix (*λ*)	1.1	0.985	0.797	0.98	1.017	1.07	1.166	1.047	0.886	0.948	1.040
Elasticity of matrix	0.616	0.62	0.762	0.896	0.875	0.852	0.844	0.877	0.882	0.926	0.897

^a^ Age‐specific values were used.

#### 2.1.1 Common starling

On the fast–slow continuum of reproductive capacity, the common starling is the fastest of the seven species we selected: It starts reproducing at an age of one year. We used the mean survival and reproductive rates for the whole Dutch breeding population (Versluijs, van Turnhout, Kleijn, & van der Jeugd, 2016), distinguishing three separate periods: 1960–1978, 1978–1990, and 1990–2012. In the first period (1960–1978), the population grew at 10% per year. This was followed by a period where the population was relatively stable (1978–1990). During the last period (1990–2012), the population declined strongly (Table 1).

#### 2.1.2 Black‐tailed Godwit

Kentie, Hooijmeijer, van der Velde, and Piersma (2017) studied two Dutch populations of the Black‐tailed Godwit in southwestern Fryslân (Skriezekrite and Kuststrook) over four to five annual transitions (Kentie et al., 2017). Godwits started reproducing at age 2, but only had 0.5–0.6 fledglings per breeding pair per year. The adults are rather long‐lived with an 86% annual survival rate. We construct separate matrix models for the two populations.

#### 2.1.3 Marsh Harrier

Mean vital rates of the Dutch breeding population of Marsh Harriers were estimated for 1997–2015 using, respectively, ring recoveries available at the Dutch Centre for Avian Migration and Demography NIOO‐KNAW and reproductive data from the Dutch Raptor Working Group (R.G. Bijlsma, unpublished data). Annual survival of Marsh Harriers was analyzed using live resightings and dead recoveries of 12,059 birds ringed as nestling between 1991 and 2016 and 74 birds ringed as “adult” in the same period (due to low sample sizes, birds ringed in their first and second calendar years were lumped with older birds in the “adult” category; H. van der Jeugd, unpublished data). Nest success was estimated using data of 1914 nests, which were followed from the beginning to the end of the nest cycle, in the Netherlands between 1997 and 2015. Based on these data, we made the assumption that these harriers start reproducing at age 3 and that in a particular year only half of the adults breed.

#### 2.1.4 Spoonbill

For each year in the 1994–2008 period, age‐specific (first‐year, second‐year, third‐year, older) annual survival rates were derived for the Dutch Spoonbill population from van der Jeugd, Ens, Versluijs, and Schekkerman (2014). Participation in the breeding population was 0% in the first 3 years and went up from 63% at age 4 to 95% at age 6 and older.

#### 2.1.5 White Stork

Schaub, Pradel, and Lebreton (2004) analyzed demographic data on White Storks in Switzerland from 1977 till 2000 (Schaub et al., 2004). Here, we extracted annual survival and reproduction rates from the COMADRE Animal Matrix Database (version 2.0.1; Salguero‐Gomez et al., 2016), but constructed matrix models with four age classes (Schaub et al., 2004). Storks start reproducing at age 3, with breeding participation increasing with age from 48% to 100%.

#### 2.1.6 Common Tern

For the Common Tern, we used mean vital rate estimates published by van der Jeugd et al. (2014) for the Dutch Waddenzee population, including the Northern part of the IJsselmeer, between 2000 and 2010 (van der Jeugd et al., 2014). The total Waddenzee and IJsselmeer population is estimated at 7,630 pairs (average population 2010–2014), constituting approximately 40% of the Dutch breeding population of about 20,000 pairs (Sovon, 2016).

#### 2.1.7 White‐tailed Eagle

Kruger, Grunkorn, and Struwe‐Juhl (2010) published demographic data on White‐tailed Eagles in Schleswig‐Holstein, Germany, over the period 1947 till 2008 (Kruger et al., 2010). Following these authors, and based on the two matrices in COMADRE v.2.0.1, we used separate matrix models for the early period (stable population dynamics) and from 1975 onwards (population growth). These eagles start reproducing at age 5.

### 2.2 Density‐independent stochastic population models (population viability analysis)

We studied the sensitivity of a single population using vital rate data presented in Table 1 to create age‐structured population models. Subsequently, we performed stochastic simulations of population dynamics: We compared a baseline with no additional mortality and scenarios with 1%, 2%, 5%, or 10% additional mortality of both juveniles and adults (i.e., mortality (m) goes to 1.01m, 1.02m, 1.05m, or 1.1m). We assume that turbine collisions are generally age‐independent because differences in visual field may underlie the cause of the interspecific differences in collision rates (Martin, Portugal, & Murn, 2012), and these are unlikely to differ between age classes. Each simulation started with the stable population structure of a matrix model parameterized with the mean vital rates of a specific species (separately per population and time period; see Table 1). Each simulated year the population vector was multiplied with a new matrix model parameterized with vital rate values randomly drawn from normal distributions (truncated if necessary to avoid negative rates or survival rates higher than 100%) based on the means and standard deviations shown in Table 1 (Boyce, 1992). As most of the pairwise correlations among vital rates were weak and not significantly different from zero (*p* > .05), we drew vital rate estimates independently (Beissinger & McCullough, 2002). Vital rate values did not depend on the size of simulated populations. However, note that the used vital rate values (Table 1) were based on measurements in the field and thus influenced by both population size and environmental factors. The initial population size was set to a thousand individuals.

Simulations ran for 100 years and were repeated a million times per scenario. Recorded statistics were the percentage change in population size after 10 years due to extra mortality. Since these statistics have a relative nature and since we simulated environmental stochasticity (sampling a matrix each year) rather than demographic stochasticity, the initial population size (1,000) had no influence on the results. Note that we could not derive PBR harvesting in these simulations because in many cases populations were declining, resulting in negative *r*_0_ values.

### 2.3 Density‐dependent population models

The consequences of wind turbine mortality for any bird population depend on that population's ability to compensate for increases in mortality rates through density‐dependent processes (Liley & Sutherland, 2007; Newton, 1998). Therefore, we also study density‐dependent population models. In general, there are two important parameters to rule the density dependence, and they are the age of first reproduction and the number of offspring per female. The first reproduction can vary greatly in a density‐dependent fashion. However, this is usually only of significance in colonially breeding, long‐lived species. In the example species that we used, density‐dependent effects on age at first reproduction are only expected in Spoonbill and Common Tern, and possibly in Black‐tailed Godwit. The data for Spoonbill were taken from the Dutch meta‐population that consists of many colonies of different ages and sizes. Lok, Overdijk, Tinbergen, and Piersma (2013), Lok, Veldhoen, Overdijk, Tinbergen, and Piersma (2017) and Oudman, de Goeij, Piersma, and Lok (2017) do not report density‐dependent effects on age at first reproduction in these Spoonbills, but did find strong evidence for negative relationships between density and fecundity (Lok et al., 2013, 2017; Oudman et al., 2017). In Common Terns, Szostek, Becker, Meyer, Sudmann, and Zintl (2014) found that colony size, but not nest density, affected reproductive output, which they take as evidence for food limitation rather than competition for nesting space, which usually results in a higher age at first reproduction (Szostek et al., 2014). In black‐tailed godwits, Kentie, Both, Hooijmeijer, and Piersma (2014) studying the same populations that we used here to derive demographic parameters showed that second calendar year individuals were not less likely to breed in high‐density areas (Kentie et al., 2014). Given these findings, we choose the number of offspring to be the parameter ruling the population growth affecting by the density.

Because we are interested in effects of increased mortality on growth and equilibrium density, we made assumptions about the intrinsic growth rate at low densities (*λ*_0_) and how the density affected the population. Because the number of offspring is often regarded as most sensitive vital rate with respect to density (Beale et al., 2006; Chase, Nur, & Geupel, 2005; Elmberg, Gunnarsson, Poysa, Sjoberg, & Nummi, 2005), we calibrated the recruitment values in each matrix to obtain *λ*_0_ values of 1.01, a slightly density‐dependent population with a growth rate of 1% per year at low density; 1.03, a density‐dependent population with a growth rate of 3% per year at low densities; and 1.1, a strong density‐dependent population with a growth rate of 10% per year at low densities. The actual density dependence was introduced by multiplying these calibrated recruitments by (1−*A*/*K*ʹ), where *K*ʹ is the number of adults where the recruitment is zero and *A* is the number of (reproducing) adults in the population. The resulting matrix models will grow to an equilibrium density (N*). We use these density‐dependent Leslie matrices to evaluate effects of extra mortality (1%, 2%, 5%, 10%) on all flying stages on the equilibrium density N* and the leftover growth rate *r*_0_, the new growth percentage at low densities after extra mortality. We calculate the response percentage of both N* and *r*_0_ for all species–population–period–*λ*_0_ combinations. Note, that also here we used randomly drawn vital rates to create matrices used for the calculations and that both response parameters are independent of *K*ʹ (the adult densities where the recruitment is zero). For the estimation of N*, we let the population grow to equilibrium, while for the estimation of *r*_0_ we studied population growth at (very) low population levels relative to *K*ʹ.

Next, we evaluated the impact of applying the potential biological removal (PBR) threshold with these density‐dependent population models. The potential biological removal is a method to assess a maximum number of animals that can be “harvested” each year without harming a population and is defined as follows (Wade, 1998):

(1) $$\text{PBR}=0.5\cdot N\cdot r_{0}\cdot F_{r}$$

In which *r*_0_ = growth rate at low densities, *N* = population estimate of the stock, and *F*_r_ = a “recovery factor” (Wade, 1998). This equation can be rearranged to the population fraction that can be harvested (*F*_p_) is as follows:

(2) $$F_{p}=\frac{\text{PBR}}{N}=0.5\cdot r_{0}\cdot F_{r}$$

In practice, this means that the harvesting fraction is scaled to the population growth rate at low densities (*r*_0_) and the recovery factor (*F*_r_). The recovery factor is a parameter between 0.1 and 1 to account for population vulnerability, the current state of the population relative to recovery goals, historic populations, or estimates of current carrying capacity. The factor is set at the low end of the range (*F*_r_ = 0.1) for species that are well below current capacity and on the high end of the range (*F*_r_ = 1) for species that are believed to be near capacity (Wade, 1998). In our Leslie matrix models, each year for each age class the PBR was calculated and subtracted from that specific age class. We used the model to explore the effects of *F*_r_ on the two response parameters N* and *r*_0_ for all species–population–period–*λ*_0_ combinations. The response of *r*_0_ is effectively the “leftover *r*_0_” with and without constant PBR harvesting. Since the classification of a population with respect to *F*_r_ is often difficult and arbitrary, we calculated the response for various *F*_r_ values along its entire range from 0.1 to 1. Also, here we used an environmental stochastic approach in which each time step the vital rates of the matrix were randomly drawn from a Gaussian curve determined by their mean and standard deviation.

## 3 RESULTS

### 3.1 Density‐independent population models

**FIGURE 2:**
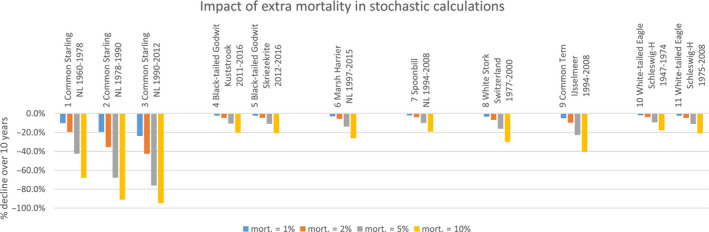
Impact of extra mortality on population reduction over 10 years in stochastic matrix models of various bird–location–period combinations. Values are % decline of populations relative to matrices without extra mortality

Comparison of the simulated scenarios showed that additional mortality has a substantial effect on population sizes and decline (Figure 2). The Common Starling was the most vulnerable species, followed by the Common Tern, whereas the White‐tailed Eagle and the Spoonbill were the least sensitive to additional mortality. For instance, 1% additional mortality to natural mortality reduced population size in 10 years between 2% and 3% in Black‐tailed Godwit, Marsh Harrier, Spoonbill, White Stork, and White‐tailed Eagle, by 5% in Common Terns, and by 10%–24% in Starlings (depending on the mean population growth rate). Five percent additional mortality reduced population sizes in 10 years by 9%–68%, with Common Starling again the most severely affected species. Ten percent additional mortality reduced population sizes in 10 years by 18%–95%.

### 3.2 Results of density‐dependent calculations

#### 3.2.1 Analyzing effects of extra mortality

**FIGURE 3:**
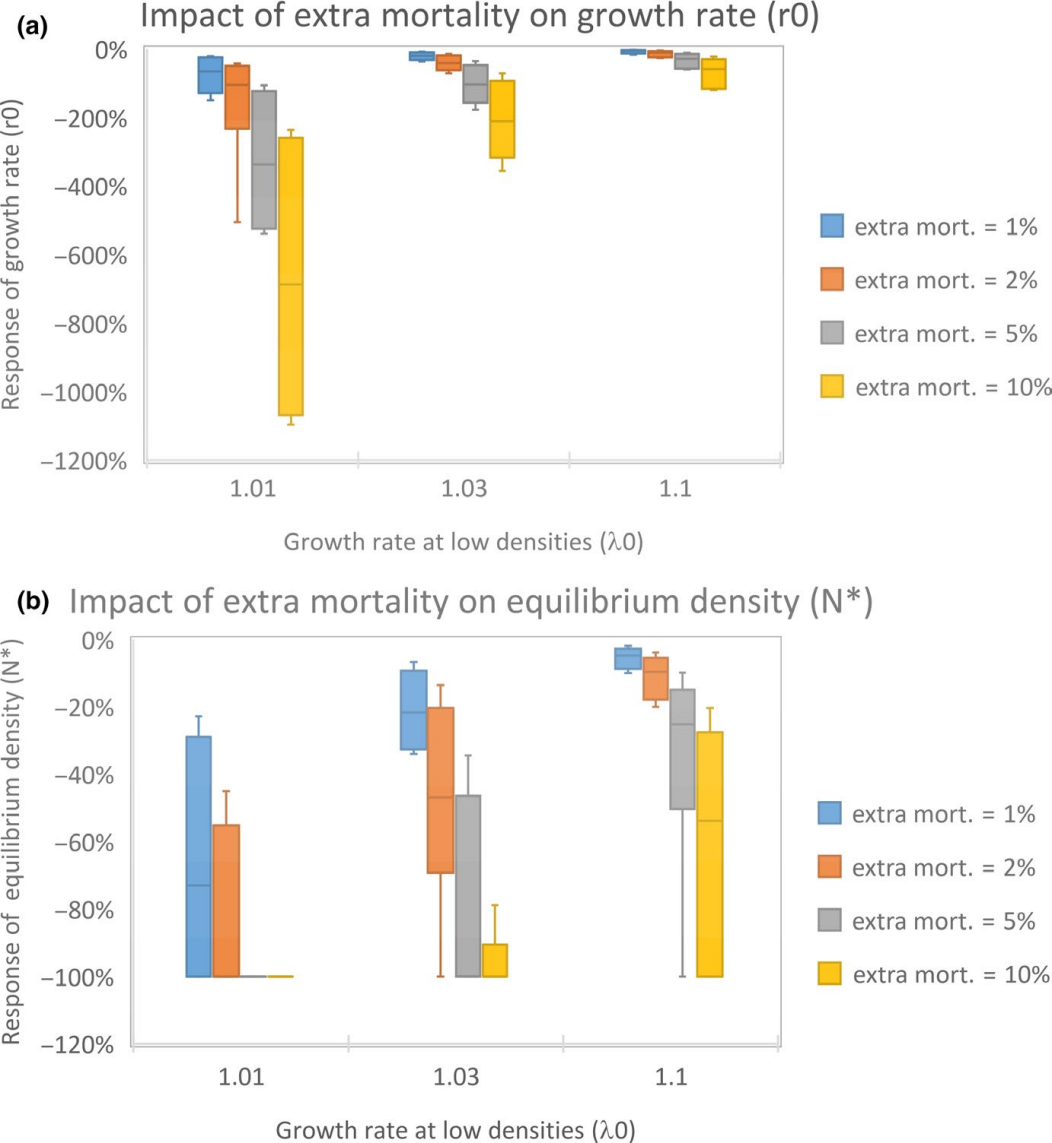
Box plots depicting the impact (percentage decline) of extra mortality on the growth rate (a) and equilibrium density (b) in bird species at various intrinsic growth rates (*λ*_0_). Quartile variation indicates the response variation between species. Species with low intrinsic growth rate (*λ*_0_) at low densities are very sensitive to extra mortality

All species–population–period–*λ*_0_ combinations had a more than proportional response of both response parameters to the 1%–10% mortality change (Table 2, Figure 3). The response was dominantly affected by the extra mortality% and the intrinsic growth rate *λ*_0_. When *λ*_0_ was low (1.01), species were more strongly affected by the extra (wind turbine) mortality than when *λ*_0_ was high (1.1). The increase in the mortality % causes an almost linear decline of leftover growth rate (*r*_0_). This was also the case for the equilibrium density N*, but here the N* could not decline below 100% since this value cannot drop below zero (Table 2, Figure 3). Note that response values of *r*_0_ with more than 100% decline and N* responses of −100% indicate populations that are extinct. Especially, populations with low *λ*_0_ and high additional mortality were prone to go extinct. Also, here the most sensitive species was the Common Starling that showed the strongest decline in the growth rate *r*_0_ due to increases wind turbine mortality between 150 and 5,200% when *λ*_0_ was 1.01 and between 16% and 589% when* λ_0_* was 1.1. Decline of growth rates *r*_0_ larger than 100% indicates a declining population, so a response of −5,200% means a decline of *r*_0_ from 0.01 to −0.51, from a slightly growing to a vastly declining population. In this species, when *λ*_0_ was 1.01, the equilibrium density dropped 100% causing extinction, and when *λ*_0_ was 1.1, the decline was between 16% and 100%. The Spoonbill, the Stork, and the White‐tailed Eagle showed the lowest sensitivity to extra mortality. Nevertheless, these species still showed a strong response to increased mortality.

**TABLE 2:** The impact of extra (wind turbine) mortality (mort.) on the resulting growth rate at low densities (*r*_0_) and the equilibrium density (N*) of various bird populations with contrasting growth rate at low densities (*λ*_0_) and age of first reproduction (AFR)

AFR	Species	Population	Period	*λ*_0_	% Response of resulting growth rate (*r*_0_)				% Response equilibrium density (N*)			
					mort. = 1%	mort. = 2%	mort. = 5%	mort. = 10%	mort. = 1%	mort. = 2%	mort. = 5%	mort. = 10%
1	Common Starling	The Netherlands	1960–1978	1.01	−150	−250	−510	−1,042	−100	−100	−100	−100
				1.03	−37	−71	−177	−356	−34	−71	−100	−100
				1.1	−16	−27	−60	−119	−10	−20	−54	−100
			1978–1990	1.01	−290	−505	−1,190	−2,460	−100	−100	−100	−100
				1.03	−100	−174	−409	−841	−100	−100	−100	−100
				1.1	−37	−61	−137	−277	−24	−51	−100	−100
			1990–2012	1.01	−610	−1,019	−2,344	−5,200	−100	−100	−100	−100
				1.03	−209	−349	−809	−1,800	−100	−100	−100	−100
				1.1	−72	−117	−267	−589	−56	−100	−100	−100
2	Black‐tailed Godwit	The Netherlands	2011–2016	1.01	−66	−133	−338	−697	−73	−100	−100	−100
		Coast		1.03	−27	−53	−136	−280	−27	−58	−100	−100
				1.1	−11	−23	−54	−113	−7	−15	−45	−100
2	Black‐tailed Godwit	The Netherlands	2012–2016	1.01	−66	−105	−336	−687	−87	−100	−100	−100
		Skriezekrite		1.03	−26	−53	−134	−276	−31	−67	−100	−100
				1.1	−9	−20	−53	−112	−7	−16	−47	−100
3	Marsh Harrier	The Netherlands	1997–2015	1.01	−43	−86	−215	−435	−29	−55	−100	−100
				1.03	−11	−22	−56	−113	−11	−22	−53	−100
				1.1	−3	−7	−16	−33	−3	−6	−15	−30
3	Spoonbill	The Netherlands	1994–2008	1.01	−21	−42	−105	−265	−23	−45	−92	−100
				1.03	−7	−14	−36	−71	−7	−14	−34	−70
				1.1	−3	−5	−12	−28	−2	−4	−10	−20
3	White Stork	Switzerland	1977–2000	1.01	−25	−51	−127	−256	−38	−73	−97	−100
				1.03	−11	−22	−54	−109	−11	−22	−59	−100
				1.1	−4	−9	−22	−44	−3	−6	−16	−34
4	Common Tern	The Netherlands	1994–2008	1.01	−107	−215	−539	−1,096	−100	−100	−100	−100
		IJsselmeer		1.03	−20	−41	−104	−211	−22	−47	−100	−100
				1.1	−6	−11	−28	−58	−5	−10	−25	−54
5	White‐tailed Eagle	Germany	1947–1974	1.01	−24	−47	−119	−240	−29	−55	−100	−100
		Schleswig‐H		1.03	−7	−15	−36	−73	−8	−15	−38	−79
				1.1	−2	−4	−11	−22	−2	−5	−15	−23
5	White‐tailed Eagle	Germany	1975–2008	1.01	−23	−46	−117	−236	−25	−49	−100	−100
		Schleswig‐H		1.03	−8	−16	−39	−79	−8	−19	−40	−81
				1.1	−3	−5	−12	−25	−2	−5	−13	−25

Note

−100% means a growth rate of 0 or an extinct population.

#### 3.2.2 Analyzing effects of using PBR thresholds

**FIGURE 4:**
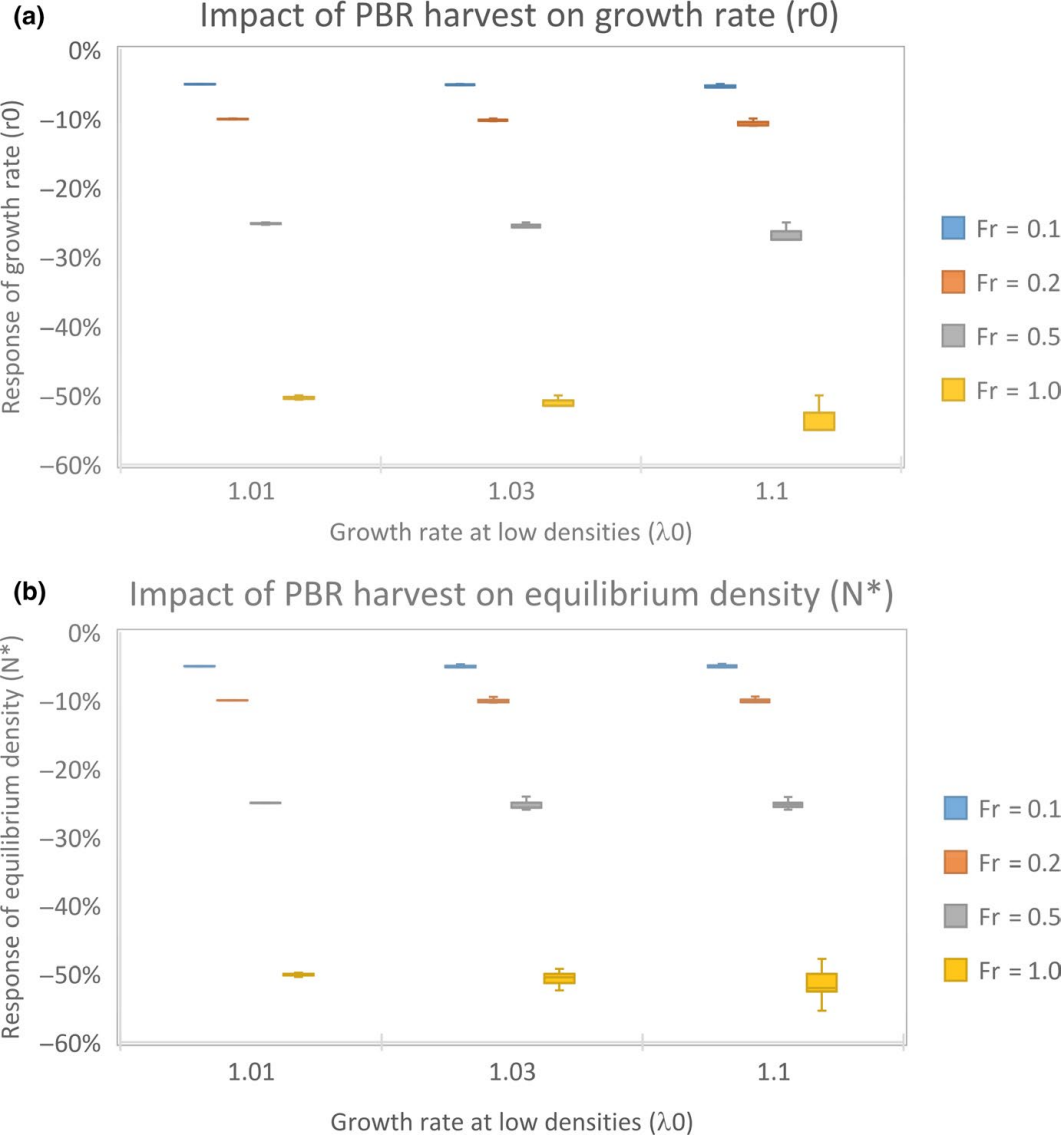
Box plots depicting the impact of PBR harvest on the growth rate (a) and equilibrium density (b) in bird species at various intrinsic growth rates (*λ*_0_). Quartile variation indicates low response variation between species, so species responses are roughly the same. The results were nearly entirely determined by the recovery factor (*F*_r_)

Exploiting a population constantly according to removals allowed by the PBR method had large consequences for the population performance parameters (Figure 4). Surprisingly, responses were rather independent on *λ*_0_ and species but were largely determined by *F*_r_. When *F*_r_ was only 0.1, we see that this removal causes an about 5% decrease in the growth rate (*r*_0_) and equilibrium density (N*). Increasing *F*_r_ up to 1.0 resulted in a 50%–55% decline in the growth rate (*r*_0_) and carrying capacity (*K*). Both N* and *r*_0_ decline almost linearly with the increase in *F*_r_. The response, *R*, of both response parameters can be approximated by the equation:

(3) $$R_{N^{*}}\approx R_{r_{0}}\approx -\frac{F_{r}}{2}$$

## 4 DISCUSSION

Acceptable additional mortality limits are guidelines for decision makers to judge whether wind turbine collisions will cause acceptable losses from a population perspective (Backes & Akerboom, 2018; Dierschke & Bernotat, 2018). Therefore, we hypothesized that the use of these limits will protect populations from significant declining. However, rather than having a negligible effect, we found a strong impact of additional mortality in the density‐independent simulations. This was especially the case for Common Starling, for which a 1% increase in mortality, that is, an additional 1% added to the assumed natural mortality (from m to 1.01m), led to an additional 10%–24% population decline after 10 years. We further showed that the use of the 5% extra mortality criterion may result in a 9%–77% population decline in ten years in the populations studied. In situations with a 10% increase in mortality, such as potentially resulting from cumulative mortality from multiple wind farms, we estimated a 17% to 95% decrease in population size in ten years. Moreover, our density‐dependent simulations showed that a 1% additional mortality can reduce the population growth rate and equilibrium density by anywhere between 2% and 610%, even causing extinction in populations with a limited growth rate (*λ*_0_). Our results thus show that the application of these widely used mortality threshold measures, such as the 1% extra mortality ORNIS criterion or other less conservative mortality limits, can severely underestimate the effect of wind turbines on bird population development.

The potential biological removal (PBR) method is often used as an alternative measure to define a level of acceptable extra mortality a population may tolerate (Green et al., 2016). Uncertainties about the outcome of PBR applications also occur as a result of the use of a “recovery factor” (Dillingham & Fletcher, 2008) which provides safety margins to PBR threshold estimates based upon expert judgment, but which often lacks any empirical validation (Green et al., 2016). We hypothesized that PBR‐based mortality will have limited effect on population performance. Our results show that in density‐dependently regulated populations, an extra mortality as allowed under PBR at *F*_r_ = 1 will cause a 50%–55% decline in the growth rate at low density *r*_0_ and the equilibrium density N*, meaning that the odds of population persistence will be strongly reduced. Perhaps surprisingly, even if the recovery factor is set at its lowest used value (*F*_r_ = 0.1), *r*_0_ and N* lose about 5% of their value. This implies that the use of the PBR method jeopardizes the population persistence of populations suffering loss from wind turbine strike, although it may not lead to extinction in populations with a positive low‐density growth rate (this method cannot be used in declining populations or in populations without density‐dependent processes). Results show that the effect of PBR‐allowed extra mortality on the population performance was largely determined by the recovery factor (*F*_r_) and was rather independent of species and growth rate (*λ*_0_). This means that the response of performance parameters following increased mortality from wind turbine collisions is nearly entirely dependent on the estimation of *F*_r_, which is a rather subjective assessment of the conservation status of a population. Presently, for sensitive populations *F*_r_ = 0.1 is often used, while in more stable populations values near one are used (Wade, 1998). We conclude that the PBR method, with *F*_r_ values between 0.1 and 1.0, is a rather blunt instrument from a population conservation point of view and only works when there is (a) growth potential (no decline) and (b) density‐dependent regulation.

We hypothesized that long‐lived species are likely to be more sensitive to an increase in mortality percentage and less able to compensate by increased reproduction (Saether & Bakke, 2000). Our results show that species with high growth rates at low densities (*λ*_0_) are indeed less sensitive to additional mortality (Table 2, Figure 3), confirming that density‐dependent processes might be responsible for some compensation of turbine mortality (Liley & Sutherland, 2007; Newton, 1998). However, short‐lived species with a high reproductive potential can be as vulnerable to similar levels of additional mortality as long‐lived species with a low reproductive potential. For example, our simulations indicated that our Common Starling population was the most vulnerable. This may seem counterintuitive given the known vulnerability to even low collision rates of various long‐lived species (de Lucas, Ferrer, Bechard, & Munoz, 2012; de Lucas, Janss, Whitfield, & Ferrer, 2008). However, additional mortality levels are reached at lower absolute number of casualties for long‐lived species that often have a (much) lower population size. In addition, the same level of additional mortality in a species with a higher mortality rate, such as Common Starling, results in a greater proportion of deaths compared with a longer‐lived species. All in all the vulnerability of the Common Starling to extra mortality is not determined by species longevity, but by the species growth rate at low density that governs the density dependence and the large stochastic variation in its vital rates.

Our results show that when applying the potential biological removal (PBR), the impacts were independent of growth rate at low densities (*λ*_0_). There was also hardly a difference between short‐lived and long‐lived species with respect to the response to PBR‐allowed removal. This is explained by the fact that the PBR estimate uses the *r*_0_ to scale the removal (Equation 1), so vulnerable species with low *r*_0_ are allowed to be less “harvested” at the same *F*_r_ value. In discrete year models, the *r*_0_ = *λ*_0_−1. So when *λ*_0_ is high, *r*_0_ is high and the PBR allowable harvest is similarly high, meaning that populations with high growth rate (*λ*_0_) can absorb a larger mortality.

Our results indicate that methods to estimate allowable mortality resulting from wind turbine collisions currently used in several northwest European countries may severely underestimate population losses in vulnerable bird populations. The mortality threshold method is based on the assumption that a relatively small increase in mortality cannot have large population impacts, but our calculations and simulations demonstrate that this is dependent on the population growth rate at low densities, *λ*_0._ When this value is low, vulnerability is high and 1% additional mortality may lead to a 23%–100% decline of the population within 10 years? Even at high values of *λ*_0_ (10% growth at low densities), our results still show a 2%–56% population decline. The PBR method accounts for the growth rate because *r*_0_ = *λ*_0_–1 (in discrete year models) and is therefore a better measure than the mortality threshold method. The allowable harvest assessed by the PBR method is largely determined by the recovery factor *F*_r_. The population impact for a vulnerable population using *F*_r_ = 0.1 is a 5% decline. However, when *F*_r_ = 1.0, as often applied for so‐called robust populations, this decline is about 50%. When used in their current form, this method allows a large potential impact on bird populations already under pressure from other anthropogenic causes. But we might reorder the PBR method by rearranging Equations (2) and (3) to estimate the harvest/mortality fraction *F*_p_ (1/year) of the population to get an acceptable response in the equilibrium density (R_N__*_):

(4) $$F_{p}\approx r_{0}*R_{N^{*}}$$

**FIGURE 5:**
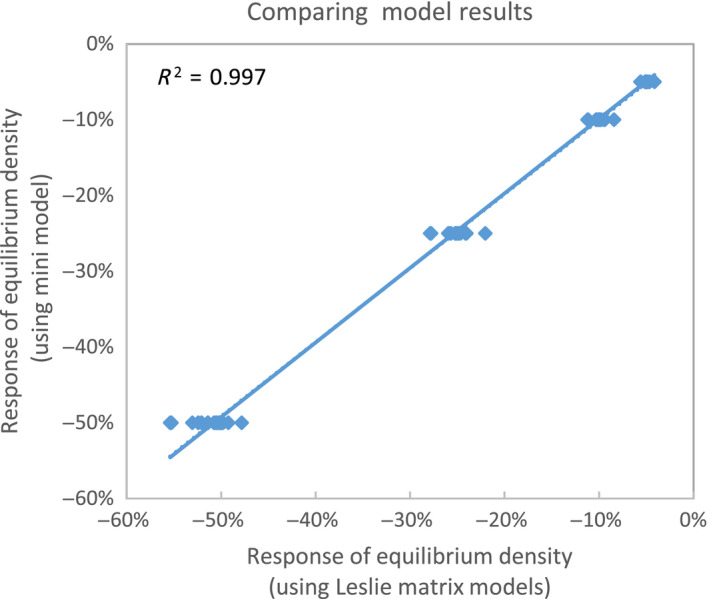
Comparing the response of equilibrium density due to fractional mortality calculated with a mini model approach summarized in equation 4 and calculated with 11 different species‐specific Leslie matrices at three growth levels and four mortality levels (*n* = 132)

In which *r*_0_ is the population growth rate at low density (*r*_0_ = *λ*_0_–1; 1/year). For instance, if the growth rate at low densities *r*_0_ = 0.1 and the acceptable population response at carrying capacity R_N__*_ = 0.01 (1% allowable decline in the population), the allowable harvest fraction *F*_p_ = 0.001 per year meaning that 0.1% of the population can be killed each year by wind turbines. This method is better than the percentage mortality and the PBR method because it directly relates population properties summarized in *r*_0_ and the allowed reduction in the equilibrium density of a population to the mortality fraction allowed and it appears robust for various matrix approaches (Figures 4and 5). Furthermore, we get rid of the rather arbitrary recovery factor (*F*_r_) used for PBR calculations. This acceptable mortality threshold method is, however, only valid when the population growth is density‐dependent. We propose this method (Equation 4) as an alternative to two commonly used threshold assessment methods.

It might be better to use more detailed approaches to evaluate wind farm impact on bird populations. Clearly, these detailed models might potentially yield more accurate results, but they need a lot of parameters and knowledge of processes. Often, the necessary data are lacking, especially on a local level, introducing many uncertainties when attempting to parameterize these models. Furthermore, these models are far more complex which makes them only usable by experts. What level of complexity to choose will depend on (a) the research question at hand and (b) available data and knowledge. Obviously, in well‐studied species it may be worthwhile to use more complex approaches. But we have to be aware that to evaluate the impact of a new wind farm on bird species in a certain area, at least several species should be evaluated. Often, there will not be enough knowledge to parameterize all these species for complex models.

Given the considerable impacts of additional mortality from wind turbine collisions on population performance of vulnerable species from wind turbine collisions (Bellebaum et al., 2013; Drewitt & Langston, 2006, 2008; Katzner et al., 2017; Schaub, 2012), it is important to consider impacts of multiple wind farms. This especially important because the number of wind farms is constantly increasing and is especially a risk for mobile species that might encounter multiple wind farms year‐round, in their breeding and foraging area. Moreover, migratory bird species might be extra vulnerable because they meet multiple turbine parks during their migration. Any estimation of impact should therefore take into account all accumulation of mortality in the populations’ year‐round range, a spatial scale reflecting the wide‐ranging movements of vulnerable, high collision‐risk species (Bellebaum et al., 2013). Despite their potential importance to evaluate the true impact of wind turbines on relevant population levels, cumulative assessments are seldom performed. Victim monitoring data are often difficult to acquire at the relevant spatial scales, and monitoring of mortality by authorities is seldom coordinated to encompass cumulative mortality at multiple sites (Drewitt & Langston, 2008). In the juridical process of some countries including the Netherlands, wind farm development projects that were completed several years back are excluded from evaluations of cumulative impact. Consequently, mortality impacts from older wind farms are ignored. For example, when the 1% mortality criterion is applied, an older wind farms are estimated to add 0.8% extra mortality, while the new wind farm is estimated to add 0.7 extra mortality. Both wind farms are admissible, whereas we are dealing with more than 1% extra mortality. When evaluating the cumulative impact, only the first wind farm is admissible.

## 5 CONCLUSIONS

We show that the use of the ORNIS 1%, the 5% mortality criterion, and potential biological removal criteria are inadequate for providing safe thresholds with respect to the impact of wind turbine collisions on populations. The responses of the population to a mortality increase are generally much higher than the mortality increase itself, whereas the PBR method is determined by the recovery factor *F*_r_. We propose a new method presented by Equation (4) as a viable alternative, provided that *r*_0_ can be estimated, is positive, and the population growth is density‐dependent. This method directly relates population properties summarized in *r*_0_ and the allowed reduction in the equilibrium density of a population to the allowed mortality fraction. Also, it appears robust for all the used matrix approaches. When a population growth is not density‐dependent or the population is declining, we propose the use of population viability analysis for more in‐depth studies of such impacts. Any additional mortality reduces the population's buffer capacity to recover from any stochastic or structural hazard, and thereby increases the risk of extinction. Particularly, those populations with low‐density dependence (low *r*_0_) are very sensitive to even a small increase in mortality. In declining populations, in which the recruitment rate is lower than the mortality rate, no “acceptable” additional mortality levels exist, as even a small increase in mortality leads to faster extinction. Finally, we should account for cumulative effects of multiple wind farms, as bird populations encounter increasing numbers of wind farms.

## CONFLICTS OF INTERESTS

None declared.

## AUTHOR CONTRIBUTION

**Peter Schippers:** Conceptualization (equal); Formal analysis (equal); Investigation (equal); Methodology (equal); Supervision (equal); Writing‐original draft (equal); Writing‐review & editing (equal). **Ralph Buij:** Conceptualization (equal); Funding acquisition (equal); Methodology (equal); Supervision (equal); Writing‐original draft (equal); Writing‐review & editing (equal). **Jana Verboom:** Conceptualization (equal); Methodology (equal); Writing‐original draft (equal); Writing‐review & editing (equal). **Alex Schotman:** Conceptualization (equal); Funding acquisition (equal); Methodology (equal). **Henk van der Jeugd:** Conceptualization (equal); Data curation (equal); Methodology (equal). **Eelke Jongejans:** Data curation (equal); Formal analysis (equal); Methodology (equal); Software (equal); Writing‐original draft (equal).

## Data Availability

The species‐specific population models constructed for this manuscript were based on previously published data, with the exception of the Marsh Harrier population model. The raw data underlying the Marsh Harrier model are made publicly available through the open repository Zenodo: http://doi.org/10.5281/zenodo.3760516 (Jongejans et al., 2020). This includes the R code and species‐specific vital rates used to construct the used matrices in this paper. The White Stork and White‐tailed Eagle population models are already part of the freely downloadable COMADRE Animal Matrix Database (https://www.compadre‐db.org/Data/Comadre), while upon publication we will send the other population models to the COMADRE team for inclusion in the database.
